# A hydrolysate of poly-*trans*-[(2-carboxyethyl)germasesquioxane] (Ge-132) suppresses Ca_v_3.2-dependent pain by sequestering exogenous and endogenous sulfide

**DOI:** 10.1016/j.redox.2022.102579

**Published:** 2022-12-15

**Authors:** Fumiko Sekiguchi, Nene Koike, Yasuhiro Shimada, Kaho Sugimoto, Hiroshi Masuda, Takashi Nakamura, Hiroaki Yamaguchi, Genzoh Tanabe, Shinsuke Marumoto, Yoshihito Kasanami, Maho Tsubota, Tsuyako Ohkubo, Shigeru Yoshida, Atsufumi Kawabata

**Affiliations:** a Laboratory of Pharmacology and Pathophysiology, Faculty of Pharmacy, Kindai University, Kowakae 3-4-1, Higashi-Osaka, 577-8502, Japan; b Asai Germanium Research Institute Co., Ltd., Suzuranoka, Hakodate, Hokkaido, 042-0958, Japan; c Yamagata University Graduate School of Medicine, Iida-nishi 2-2-2, Yamagata, 990-9585, Japan; d Department of Pharmacy, Yamagata University Hospital, Iida-nishi 2-2-2, Yamagata, 990-9585, Japan; e Laboratory of Pharmaceutical Organic Chemistry, Faculty of Pharmacy, Kindai University, 3-4-1 Kowakae, Higashi-Osaka, 577-8502, Japan; f Joint Research Center, Kindai University, 3-4-1 Kowakae, Higashi-Osaka, 577-8502, Japan; g Division of Basic Medical Sciences and Fundamental Nursing, Faculty of Nursing, Fukuoka Nursing College, Fukuoka, 814-0193, Japan; h Department of Life Science, Faculty of Science and Engineering, Kindai University, 3-4-1 Kowakae, Higashi-Osaka, 577-8502, Japan

**Keywords:** Organogermanium, Ge-132, Sulfide, Ca_v_3.2 T-type calcium channel, Zinc, Pain, CPA, cyclophosphamide, CSE, cystathionine-γ-lyase, H_2_S, hydrogen sulfide, ^1^H NMR, proton nuclear magnetic resonance, MS/MS, tandem mass spectrometry, T-channel, T-type Ca^2+^ channel, THGP, 3-(trihydroxygermyl)propanoic acid

## Abstract

Poly-*trans*-[(2-carboxyethyl)germasesquioxane] (Ge-132), an organogermanium, is hydrolyzed to 3-(trihydroxygermyl)propanoic acid (THGP) in aqueous solutions, and reduces inflammation, pain and cancer, whereas the underlying mechanisms remain unknown. Sulfides including H_2_S, a gasotransmitter, generated from l-cysteine by some enzymes including cystathionine-γ-lyase (CSE), are pro-nociceptive, since they enhance Ca_v_3.2 T-type Ca^2+^ channel activity expressed in the primary afferents, most probably by canceling the channel inhibition by Zn^2+^ linked via coordinate bonding to His^191^ of Ca_v_3.2. Given that germanium is reactive to sulfur, we tested whether THGP would directly trap sulfide, and inhibit sulfide-induced enhancement of Ca_v_3.2 activity and sulfide-dependent pain in mice. Using mass spectrometry and ^1^H NMR techniques, we demonstrated that THGP directly reacted with sulfides including Na_2_S and NaSH, and formed a sulfur-containing reaction product, which decreased in the presence of ZnCl_2_. In Ca_v_3.2-transfected HEK293 cells, THGP inhibited the sulfide-induced enhancement of T-type Ca^2+^ channel-dependent membrane currents. In mice, THGP, administered systemically or locally, inhibited the mechanical allodynia caused by intraplantar Na_2_S. In the mice with cyclophosphamide-induced cystitis and cerulein-induced pancreatitis, which exhibited upregulation of CSE in the bladder and pancreas, respectively, systemic administration of THGP as well as a selective T-type Ca^2+^ channel inhibitor suppressed the cystitis-related and pancreatitis-related visceral pain. These data suggest that THGP traps sulfide and inhibits sulfide-induced enhancement of Ca_v_3.2 activity, leading to suppression of Ca_v_3.2-dependent pain caused by sulfide applied exogenously and generated endogenously.

## Highlights

• Sulfide enhances Ca_v_3.2 activity by interacting with Zn^2+^ linked to Ca_v_3.2.
• THGP, an organogermanium compound hydrolysate, directly reacts with sulfide.
• THGP inhibits the sulfide-induced enhancement of Ca_v_3.2 activity.
• THGP suppresses the Ca_v_3.2-depedent pain caused by sulfide in mice.
• The organogermanium is useful to treat sulfide-induced symptoms including pain.

## Graphical abstract

**Figure:**
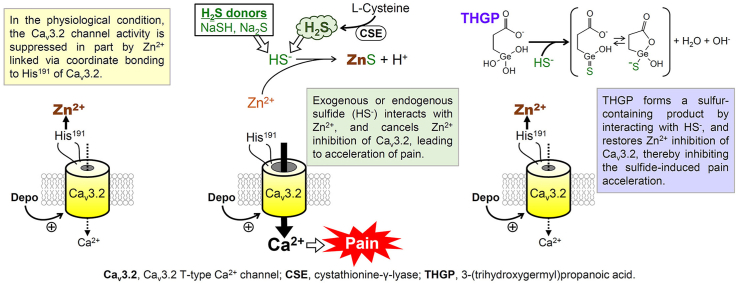

## 1 Introduction

**Fig. 1:**
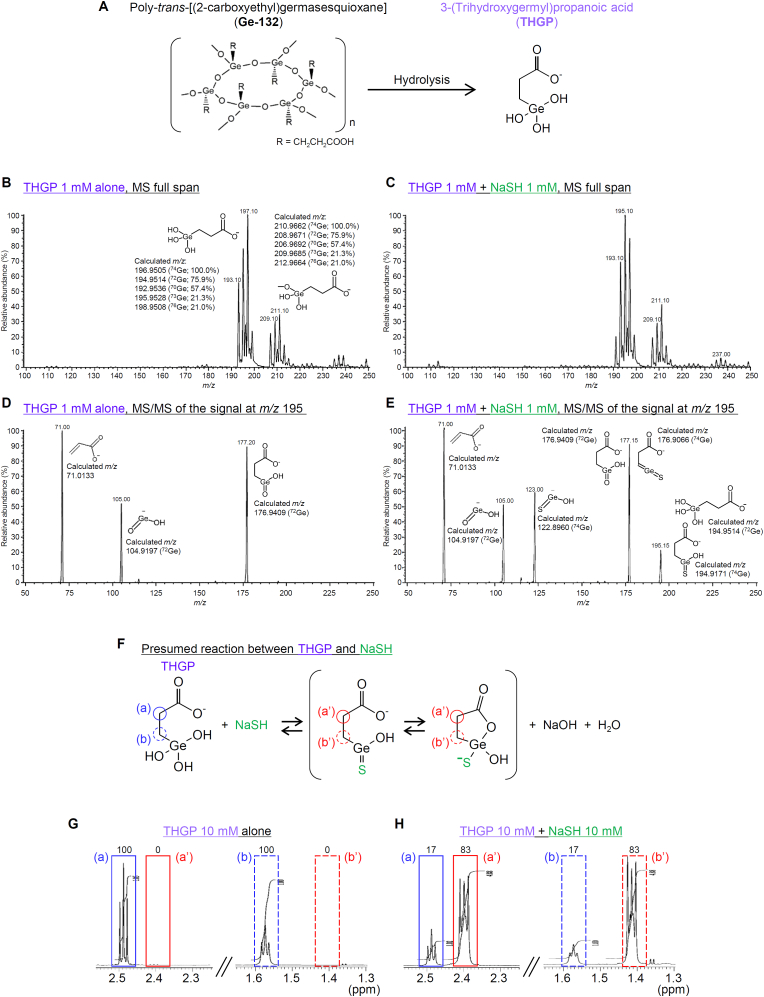
Presumed structure of the reaction product of THGP, a hydrolysate of Ge-132, and NaSH, as estimated by MS/MS and ^1^H NMR analyses. (A) Structure of Ge-132 and its hydrolysate, THGP. (B, C) Ion mass spectra of THGP (B) and the mixture of THGP and NaSH (C), provided by MS analysis. NaSH and THGP, each at a final concentration of 1 mM, were mixed at room temperature, and were subjected to the MS analysis. (D, E) Product ion mass spectra provided by MS/MS analysis of the precursor signals at *m/z* 195 in THGP at 1 mM alone (D) and the mixture of THGP at 1 mM and NaSH at 1 mM (E) with a collision energy of −20 V. (F) Presumed chemical reaction formula for the mixture of THGP and NaSH. (a) and (b) in THGP, and (a’) and (b’) in the presumed generated compound indicate two methylene groups adjoining the carbonyl group and germanium atom, respectively. (G, H) ^1^H NMR spectra of THGP (G) at 10 mM alone and the mixture of THGP and NaSH, each at 10 mM (H), as measured at 800 MHz. Signals of two methylene groups, (a) and (b), in THGP appeared at *δ*_H_ 2.49 and 1.57, respectively, (G), and once mixed with NaSH, shifted to higher magnetic fields, i.e., *δ*_H_ 2.39 and 1.42, that probably reflected the corresponding two methylene groups, (a') and (b'), respectively, in the presumed generated compound (H).

In contrast to inorganic germanium that accumulates in the kidney and is hazardous to mammals, organogermanium compounds, such as poly-*trans*-[(2-carboxyethyl)germasesquioxane] (Ge-132), and 3-oxygermylpropionic acid polymer, i.e. propagermanium, are considered avirulent and have therapeutic potentials against some diseases including cancer and chronic hepatitis [[1], [2], [3], [4]]. Ge-132 is hydrolyzed to 3-(trihydroxygermyl)propanoic acid (THGP) (Fig. 1A) in aqueous solutions [5], and shows immunostimulatory [[6], [7], [8]], anti-inflammatory [9], anti-cancer [[10], [11], [12]], analgesic [13] and anti-oxidant [14] activities. However, the molecular mechanisms for the widespread biological activities of Ge-132 largely remain unclear. Most interestingly, a series of studies employing nuclear magnetic resonance (NMR) analysis have demonstrated that THGP, the hydrolysate of Ge-132, forms complexes with *cis*-diol compounds including saccharides, nucleotides and 3,4-dihydroxy-l-phenylalanine (l-DOPA) [5,15,16], which might, in part, interpret pharmacological activities of Ge-132.

We have shown that sulfides including hydrogen sulfide (H_2_S), a gasotransmitter, are capable of enhancing the activity of Ca_v_3.2 isoform of T-type Ca^2+^ channels (T-channels), and cause somatic and visceral pain or hypersensitivity in mice and rats, an effect abolished by knockout or knockdown of Ca_v_3.2 gene and by T-channel blockers [[17], [18], [19], [20], [21], [22], [23], [24]]. The mechanisms by which sulfide enhances Ca_v_3.2 T-channel activity have yet to be directly clarified. Nonetheless, considering the high affinity of sulfides to Zn^2+^, it is likely that, as does l-cysteine [25,26], sulfides might interact with Zn^2+^ linked by coordinate bonding to a histidine residue at position 191 (His^191^) in the second extracellular loop of domain I of Ca_v_3.2, and cancel the Zn^2+^ inhibition of Ca_v_3.2 activity. There is also plenty of evidence that endogenous H_2_S produced from l-cysteine by cystathionine-γ-lyase (CSE), an H_2_S-forming enzyme, contributes to the Ca_v_3.2-dependent pain in various animal models for inflammatory and neuropathic somatic pain [17,27,28], as well as visceral pain associated with cystitis or pancreatitis [19,[29], [30], [31], [32]]. Interestingly, there is evidence that germanium reacts with chalcogens including sulfur [33,34] and that Ge-132 relieves pain in cancer patients at a terminal stage [35]. We thus assumed that THGP, the hydrolysate of Ge-132, might directly trap sulfide, a pronociceptive molecule, resulting in pain suppression. To test this hypothesis, in the present study, we examined whether THGP would react with sulfide *in vitro* and inhibit sulfide-induced enhancement of T-channel-dependent currents in Ca_v_3.2-transfected HEK293 cells. Further, we investigated the effects of THGP on exogenously applied sulfide-induced paw allodynia, which is dependent on Ca_v_3.2 [17,18,21,23], and on visceral pain accompanying cystitis and pancreatitis, known to involve the endogenous sulfide/Ca_v_3.2 pathway [19,[29], [30], [31], [32]], in mice.

## 2 Materials and methods

### 2.1 Chemicals

Poly-*trans*-[(2-carboxyethyl)germasesquioxane] (Ge-132), was synthesized in Asai Germanium Research Institute Co., Ltd. (Kawasaki, Japan). Cyclophosphamide (CPA) and sodium sulfide (Na_2_S) were purchased from Sigma-Aldrich (St. Louis, MO, USA). Zinc chloride (ZnCl_2_), sodium hydrogen sulfide (NaSH), TTA-A2 and cerulein were purchased from Kishida Chemicals Co., Ltd. (Osaka, Japan), Strem Chemicals, Inc. (Newburyport, MA, USA), Alomone Labs. (Jerusalem, Israel) and Bachem (Bubendorf, Switzerland), respectively. THGP, NaSH and Na_2_S were dissolved in ultra-pure water for the MS/MS and whole-cell patch clamp experiments, and in phosphate buffer (20 mM, pH 7.4) or Tris-HCl buffer (1 M, pH 7.4) prepared with D_2_O instead of H_2_O for ^1^H NMR analysis. For administration to mice, THGP, NaSH, Na_2_S, CPA and cerulein were dissolved in saline, and TTA-A2 was in 0.5% methyl cellulose containing 1.3% DMSO and 10% Tween-80.

### 2.2 Tandem mass spectrometry analysis

NaSH at 1 mM and THGP at 1 mM were mixed and incubated for 10 min at room temperature, and the structure of the reaction products was analyzed by tandem mass spectrometer, LCMS-8060 (Shimadzu, Kyoto, Japan), equipped with heated ESI probe. The pH of the mixture was adjusted to 7.0 with NaOH. Mass spectrometry (MS) and tandem mass spectrometry (MS/MS) were carried out in the negative ion mode. The samples were analyzed by flow injection analysis. The mobile phase consisted of water/methanol (1:2, v/v) and it was pumped at a flow rate of 0.2 mL/min. The overall run time was 1 min. The injection volume was 2 μL. Probe voltage was set at −3000 V, DL temperature was 150 °C and 250 °C, heat block temperature was 400 °C, and interface temperature was 100 °C. Nebulizer gas flow was 3 L/min, drying gas flow was 10 L/min, and heating gas flow was 10 L/min. ESI mass spectra for reaction products were measured at a scan range from *m/z* 100 to 250. The ion at *m/z* 195 in the negative ion full-scan mass spectra obtained by MS analysis of the mixture of THGP and NaSH was selected as a precursor for MS/MS analysis, since the relative signal at *m/z* 195 in the mixture of THGP and NaSH was greater than that in THGP alone. Product ion spectra were measured at a scan range from *m/z* 50 to 250 with the collision energy of −20 and −35 V. Data were acquired and analyzed using LabSolutions LCMS software (Shimadzu). The values of calculated *m/z* were determined using ChemBioDraw Ultra 11.0 (PerkinElmer, Inc., Waltham, MA, USA).

### 2.3 Proton nuclear magnetic resonance analyses of the interaction between THGP and sulfides

In proton nuclear magnetic resonance (^1^H NMR) analysis, a phosphate buffer solution (20 mM, pH 7.4) was prepared with D_2_O and used for preparing samples. The solution of THGP at a final concentration of 10 mM was mixed with NaSH at 10 mM or Na_2_S at 1.25–10 mM, 10 min (or 5 min in some experiments) before beginning the NMR analysis. In the experiments to study the reaction between THGP and Na_2_S, each at 10 mM, in the presence of ZnCl_2_ at 0.1–10 mM, a Tris-HCl buffer solution (1 M, pH 7.4) instead of the phosphate buffer were prepared using D_2_O and deuterium chloride solution, in order to avoid the formation of Zn_3_(PO_4_)_2_ precipitation. The deposited ZnS in the reaction mixture was filtered off immediately before NMR analysis. ^1^H NMR spectrum of the reaction mixtures or its filtrates was measured at 25 °C by a JEOL JNM-ECA 800 (800 MHz, ^1^H).

### 2.4 Cell culture

HEK293 cells that stably express human Ca_v_3.2 T-channels (hCa_v_3.2-HEK293) [23,24,36] were cultured in Dulbecco's modified Eagle's medium (DMEM) with l-glutamine (Wako Pure Chem. Inc. Ltd., Osaka, Japan) including 10% fetal calf serum (FCS) (Nichirei Biosci. Inc., Tokyo, Japan), 100 unit/mL penicillin, 100 μg/mL streptomycin (Gibco, Carlsbad, CA, USA), 0.2 mg/mL G418 (Sigma-Aldrich). For the whole-cell patch-clamp recording, the cells (10^4^ cells/35-mm dish) were cultured in the above-mentioned medium for 4 h, and thereafter, in the FCS-free medium overnight for the use in the electrophysiological experiments.

### 2.5 Measurement of T-currents by the whole-cell patch-clamp recording

T-channel-dependent Ba^2+^ currents (T-currents) were measured by the whole cell-patch clamp recording in hCa_v_3.2-HEK293 cells, as described elsewhere [23,24,37]. The composition of the extracellular solution (mM) was: 152 tetraethylammonium (TEA)-Cl, 10 BaCl_2_ and 10 HEPES, adjusted to pH 7.4 with TEA-OH. The composition of the intracellular solution (mM) was: 110 Cs–MeSO_4_, 14 creatine phosphate, 10 HEPES, 9 EGTA, 5 Mg-ATP, and 0.3 Tris-GTP, adjusted to pH 7.2 with CsOH. The resistance of the patch electrodes was a range of 3–5 MΩ. Series-resistance was compensated by 80%, and current recordings were low-pass filtered (<5 kHz). The cell membrane voltage was held at −80 mV, and T-currents were elicited every 12 s by a test pulse of 200 ms duration at −20 mV. The cells were superfused at a rate of 3 mL/min with the extracellular solution, and after stabilized, exposed to the solution containing THGP (1–10 mM) for 5 min, and then to the solution containing Na_2_S at 10 μM in addition to THGP for 10 min.

### 2.6 Animals

Female (for the cystitis model) or male (other experiments) ddY mice (18–35 g) were purchased from Kiwa Laboratory Animals Co. Ltd. (Wakayama, Japan). The mice were housed in a temperature-controlled room at 24 °C under a 12-h day/night cycle, and had free access to food and water. All animals were used with approval by Kindai University's Committee for the Care and Use of Laboratory Animals, and all procedures employed in the present study were in accordance with the guidelines of the Committee for Research and Ethical Issues of IASP (www.iasp-pain.org/Education/Content.aspx?ItemNumber=1217).

### 2.7 Assessment of mechanical allodynia induced by intraplantar injection of Na_2_S in mice

Mice were placed on a risen wire mesh floor, covered with a clear plastic box (10 × 10 × 10 cm) and habituated to the experimental environment. Then, the mid-plantar surface of the right hindpaw was stimulated with von Frey filaments (0.008, 0.02, 0.04, 0.07, 0.16, 0.4, 0.6 and 1.0 g), and 50% paw withdrawal threshold was determined according to the up-down method [23,38]. After measurements of baseline thresholds, mice received intraplantar (i.pl.) injection of Na_2_S at 10 pmol/paw in a volume of 10 μL in the right hindpaw, and thereafter, the nociceptive threshold was measured every 15 min. THGP at 0.02, 0.2 and 2 μg/paw (0.1, 1 and 10 nmol/paw, respectively) was co-injected i.pl. with Na_2_S 10 pmol/paw, and THGP at 30 and 100 mg/kg (150 and 500 μmol/kg) was administered i.p. 30 min before i.pl. Na_2_S at the same dose.

### 2.8 Creation of a cyclophosphamide-induced cystitis model and assessment of bladder pain-like nociceptive behavior, referred hyperalgesia, bladder swelling and micturition in mice

The model of cyclophosphamide (CPA)-induced cystitis in mice was prepared according to the previously reported method [[39], [40], [41]] with minor modifications, in which the increased dose, 400 mg/kg, but not 300 mg/kg, of CPA was used to induce highly reproducible bladder pain-like behavior and referred hyperalgesia in mice [30,42]. The bladder pain-like nociceptive behavior, such as licking the skin region of the bladder and pressing the lower abdomen against the floor, was counted for a 30-min period starting 3.5 h after i.p. CPA, and subsequently, the referred hyperalgesia was evaluated by stimulating the skin region between the anus and urethral opening with each of four distinct von Frey filaments (0.008, 0.07, 0.4 and 1.0 g) [41]. Thereafter, micturition frequency was measured by a voiding spot method for 2 h [32,43]. Finally, i.e. approximately 6.5 h after i.p. CPA, the mice were killed by cervical dislocation, and the bladder was isolated for determination of the wet tissue weight as a marker of bladder swelling and for Western blot analysis of CSE protein levels. THGP at 30 and 100 mg/kg or TTA-A2, a selective T-channel blocker, at 1 mg/kg was administered i.p. 3 h after i.p. CPA.

### 2.9 Creation of a cerulein-induced pancreatitis model and assessment of referred hyperalgesia and plasma amylase activity in mice

The pancreatitis model was created in male mice by repetitive i.p. injection of cerulein at 50 μg/kg at 1-h intervals, 6 times in total, as described previously [19,44]. Referred hyperalgesia was assessed by using four distinct von Frey filaments (0.02, 0.07, 0.16 and 1.0 g), 5.5, 6.0 and 6.5 h after the onset of cerulein injection. Thereafter, i.e. 7 h after the onset of cerulein injection, citrated blood was withdrawn from the aorta of mice anesthetized with i.p. injection of midazolam at 4 mg/kg, medetomidine 0.3 mg/kg and pentobarbital 10 mg/kg, to determine the plasma amylase activity as an indicator of the severity of the evoked pancreatitis, and the pancreas was excised afterwards for determination of tissue weight as a marker of pancreatic edema and for Western blot analysis of CSE protein levels in the pancreatic tissue. THGP at 100 mg/kg or TTA-A2 at 1 mg/kg was administered i.p. 5 min after the final (6th) injection of cerulein.

### 2.10 Western blot analysis

Protein expression levels of CSE was analyzed by Western blotting in the isolated bladder and pancreas of mice, as described previously [19,29]. The primary antibodies employed were an anti-CSE rabbit antibody (sc-135203, Santa Cruz Biotechnol., Santa Cruz, CA, USA) and an anti-GAPDH rabbit antibody (sc-25778, Santa Cruz Biotechnol.). A HRP-conjugated anti-rabbit IgG (Cell Signaling Technol., Beverly, MA, USA) was used as a secondary antibody. Immunopositive bands were developed by Chemi-Lumi One Super (Nacalai Tesque, Kyoto, Japan), and quantified using densitometric software (ImageJ 1.44p, http://imagej.nih.gov/ij).

### 2.11 Statistics

Data are represented as the mean ± S.E.M. Statistical significance for parametric data were analyzed by an analysis of variance followed by the Tukey's test for multiple comparisons or Student's *t*-test for two-group data. For non-parametric analyses, Kruskal-Wallis *H*-test followed by a least significant difference-type test was employed for multiple comparisons. Significance was set at a level of p < 0.05.

## 3 Results

### 3.1 THGP directly interacts with sulfide *in vitro*

To test the possible direct interaction of THGP, the hydrolysate of Ge-132 (Fig. 1A), with sulfide, we analyzed the structure of THGP alone and the mixture of THGP and NaSH, using the MS/MS system. In MS analysis of THGP alone at 1 mM, the negative ion full-scan mass spectra showed five major signals at *m/z* 193, 195, 196, 197 and 199, corresponding to the calculated *m/*z values of THGP containing five different major natural isotopes of germanium, ^70^Ge, ^72^Ge, ^73^Ge, ^74^Ge and ^76^Ge, respectively (Fig. 1B). The relative abundance of the signal at *m/z* 197 for ^74^Ge-containing THGP (calculated *m/z*: 196.9505) was the greatest (Fig. 1B), in agreement with the previous report from our group [45]. It is to be noted that additional five minor signals were detected at *m/z* 207, 209, 210, 211 and 213 (Fig. 1B), as seen in the previous report [45], which might indicate the existence of methylated products of THGP (Fig. 1B) possibly due to the reaction with methanol present in the mobile-phase. Next, in MS analysis of the mixture of THGP at 1 mM and NaSH at 1 mM, the relative abundance of the signal at *m/z* 195 was the greatest in the negative ion full-scan mass spectra (Fig. 1C), differing from the results of the MS analysis of THGP alone (Fig. 1B), thereby indicating that the increased relative signal at *m/z* 195 reflects generation of a reactant between THGP and NaSH, in addition to ^72^Ge-containing THGP itself (see Fig. 1B). Thus, the signal at *m/z* 195 in THGP alone and the mixture of THGP and NaSH was selected as a precursor for MS/MS analysis. In MS/MS analysis of THGP alone with a collision energy of −20 V, the collision-induced dissociation product ions of the precursor signal at *m/z* 195 corresponding to ^72^Ge-containing THGP were detected at *m/z* 177 [dehydrated product of THGP, calculated *m/z*: 176.9409 (^72^Ge)], 105 [O=Ge^−^-OH, calculated *m/z*: 104.9197 (^72^Ge)] and 71 (CH_2_=CH–COO^-^, calculated *m/z*: 71.0133) (Fig. 1D). The MS/MS analysis of THGP alone with a greater collision energy, −35 V, provided only two product ions at *m/z* 105 and 71 (Fig. S1A), being consistent with the profile of MS/MS analysis of the precursor signal at *m/z* 197 corresponding to ^74^Ge-containing THGP in the previous report [45]. On the other hand, in MS/MS analysis of the mixture of THGP and NaSH with a collision energy of −20 V, the collision-induced dissociation product ions of the precursor signal at *m/z* 195 were detected at *m/z* 195, 177, 123, 105 and 71 (Fig. 1E). It is noteworthy that the dissociation product ions at *m/z* 195 and 123 were not detectable in the MS/MS analysis of the precursor signal at *m/z* 195 for ^72^Ge-containing THGP alone. The signals at *m/z* 195, 177 and 123 corresponded to calculated *m/z* 194.9171, 176.9066 and 122.8960 (^74^Ge), respectively, of the expected collision-induced dissociation product ions of a possible sulfur-containing reactant in the mixture of THGP and NaSH. The MS/MS analysis of the mixture of THGP and NaSH with a greater collision energy, −35 V, provided a collision-induced dissociation product ion at *m/z* 123 corresponding to the calculated *m/z* 122.8960 (^74^Ge) of the dissociation product ion of the sulfur-containing reactant, in addition to the signals at *m/z* 105 and 71 detected in MS/MS analysis of THGP alone (Fig. S1B). Collectively, the mixing of THGP and NaSH appears to generate a chemical in which two hydroxyl groups on a germanium (Ge) atom of THGP are replaced with a sulfur atom (Fig. 1F).

**Fig. 2:**
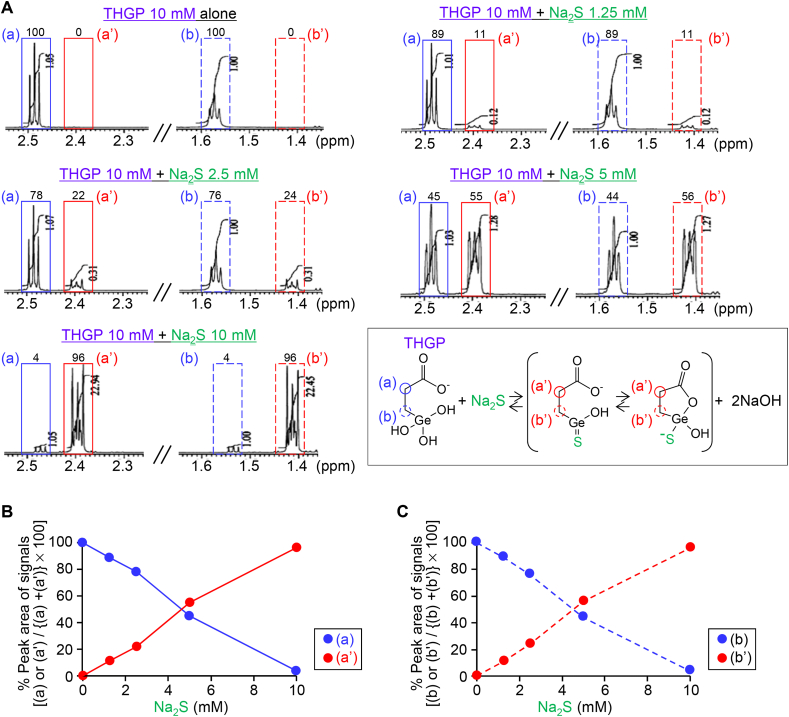
Increased formation of the sulfur-containing reaction product by the interaction of THGP with increased Na_2_S concentrations, as assessed by the ^1^H NMR analysis. ^1^H NMR spectra of the mixture of THGP at 10 mM and Na_2_S at 0–10 mM were measured at 800 MHz. (A) The signals of two methylene groups adjoining the carbonyl group and germanium atom, (a') and (b'), respectively, in the presumed sulfur-containing reaction product increased depending on Na_2_S concentrations, and conversely, the signals of the corresponding two methylene groups, (a) and (b), in THGP decreased. (B, C) Changes in % peak areas of signals (a) and (a’) (B) and of signals (b) and (b’) (C) in the mixture of THGP at 10 mM and Na_2_S at 0–10 mM.

Next, we analyzed the structure of compounds present in the mixture of THGP and NaSH using a ^1^H NMR technique. In the NMR spectrum of THGP at 10 mM alone, two proton triplet signals were observed at *δ*_H_ 2.48 (a) and 1.57 (b), indicating two methylene groups adjoining a carbonyl group and a Ge atom, respectively (Fig. 1F and G). On the other hand, in the NMR spectrum of the mixture of THGP and NaSH, each at a final concentration of 10 mM, as assessed after 10-min reaction at room temperature, the two proton triplet signals, (a) and (b), of THGP shifted to the higher magnetic fields, i.e. *δ*_H_ 2.40 (a’) and 1.42 (b’), respectively, indicating that a large proportion (83%) of THGP changed to the sulfur-containing reaction product through the reaction with sulfide (Fig. 1F, H). It is noteworthy that NMR analysis performed even 5 min after mixing THGP and NaSH provided almost the same results, indicating that the reactions between THGP and NaSH were very fast and quickly reached an equilibrium. We also confirmed such interaction between THGP and Na_2_S, another sulfide compound. THGP at 10 mM was mixed with Na_2_S at 1.25, 2.5, 5 or 10 mM and incubated at room temperature for 10 min. The NMR spectrum of the mixture of THGP and Na_2_S showed that two proton triplet signals, (a) and (b), shifted to the higher magnetic fields, (a’) and (b’), respectively, in a manner depending on concentrations of Na_2_S (Fig. 2A). The % peak areas of (a’) and (b’) signals increased depending on Na_2_S concentrations, and conversely, (a) and (b) signals decreased (Fig. 2B and C). For instance, it is roughly estimated that, when 10 mM THGP is mixed with 5 and 10 mM sulfide, approximately 50% and 95% of THGP change to the sulfur-containing reaction product, respectively (Fig. 2B and C). We also confirmed that the above-mentioned NMR profiles remained constant even when the incubation time of the mixture of THGP and Na_2_S was prolonged overnight, indicating that the reaction between THGP and Na_2_S is reversible and quickly reaches an equilibrium. Collectively, THGP may be able to trap sulfide that possibly interact with Zn^2+^ linked via coordinate bonding to His^191^ of Ca_v_3.2, which inhibits the channel activity [46].

### 3.2 The reaction sensitivity of THGP to sulfide in the presence of Zn^2+^

**Fig. 3:**
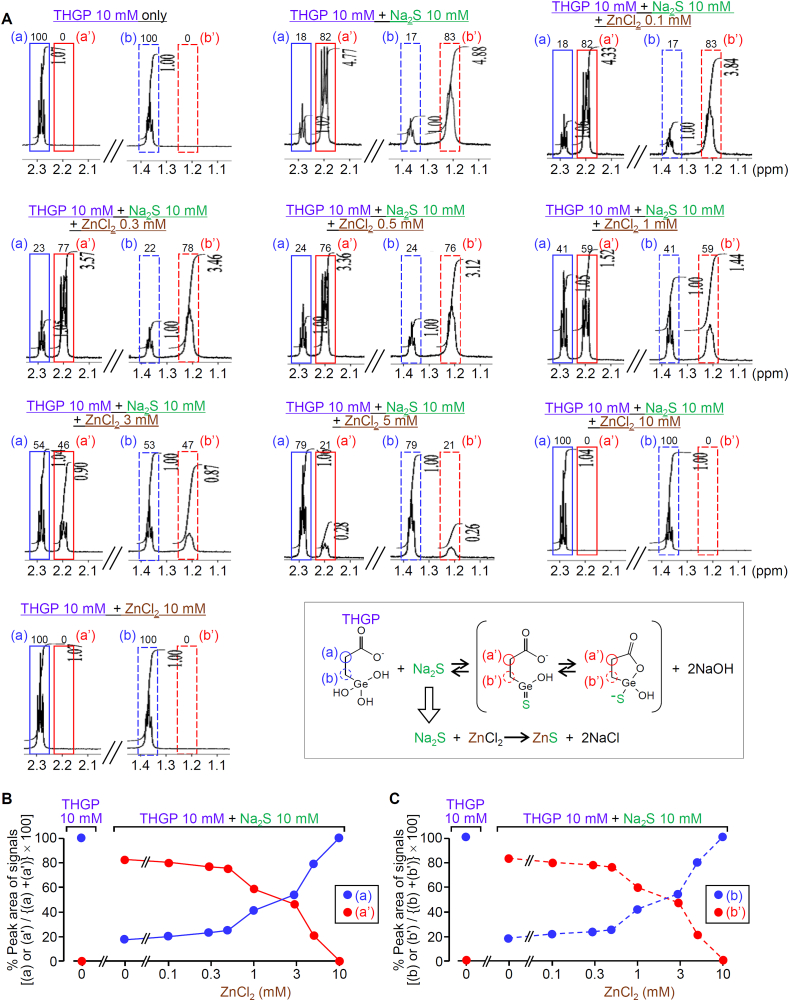
Reduced formation of the sulfur-containing reaction product of THGP and Na_2_S in the presence of ZnCl_2_. ^1^H NMR spectra of THGP alone and the mixture of THGP at 10 mM and Na_2_S at 10 mM in the presence of ZnCl_2_ at 0–10 mM were measured at 800 MHz. (A) The increased signals, (a') and (b'), and the decreased signals, (a) and (b), in the mixture of THGP and Na_2_S, each at 10 mM, were reduced by ZnCl_2_ in a concentration-dependent manner. (B, C) Concentration-dependent inhibition by ZnCl_2_ of the changes in % peak areas of signals of (a) and (a') (B) or (b) and (b') (C) in the mixture of THGP and Na_2_S, each at 10 mM.

It is essential to compare the relative affinities of THGP and Zn^2+^ to sulfide, if we use THGP to inhibit the interaction of sulfide with Zn^2+^ linked to His^191^ of Ca_v_3.2. Thus, we examined the reaction sensitivity of THGP to Na_2_S in the presence of Zn^2+^ at different concentrations. It is to be noted that a Tris-HCl buffer instead of the phosphate buffer was used to avoid the formation of Zn_3_(PO_4_)_2_ precipitation in this experiment. Under this condition, 82–83% of 10 mM THGP, once mixed with 10 mM Na_2_S in the absence of ZnCl_2_, changed to the sulfur-containing reaction product, as estimated from changes in % peak area of triplet signals from (a) and (b) to (a’) and (b’), respectively (Fig. 3A, top center, and Fig. 3B and C). This reaction between THGP and sulfide decreased by addition of ZnCl_2_ in a concentration-dependent manner (Fig. 3), e.g. ZnCl_2_ at 3 and 10 mM decreased the % peak areas of (a’) and (b’) signals of the 10 mM THGP/10 mM Na_2_S mixture from 82-83% to 46–47% and 0%, respectively, and conversely, (a) and (b) signals increased (Fig. 3).

### 3.3 Inhibitory effect of THGP on the Na_2_S-induced enhancement of T-currents in Ca_v_3.2-transfected HEK293 cells

**Fig. 4:**
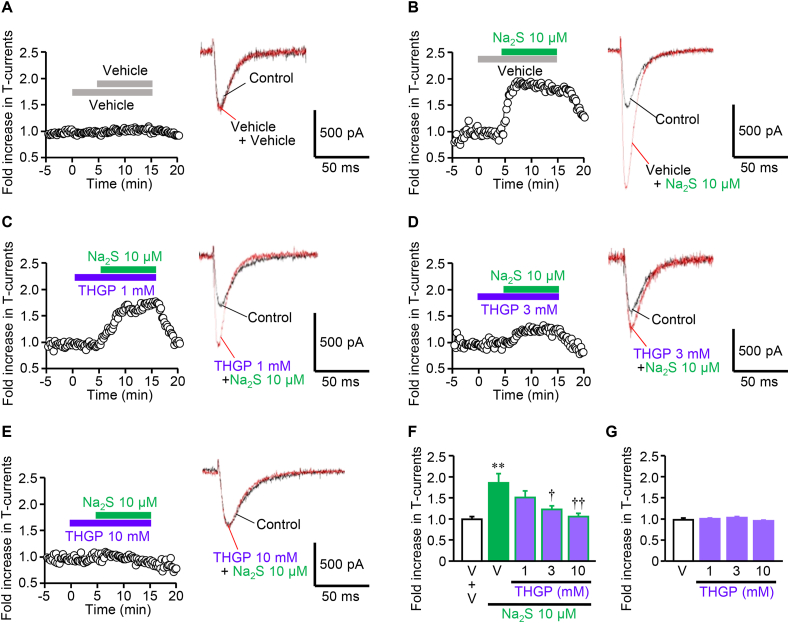
Inhibitory effect of THGP on the Na_2_S-induced increase in T-channel-dependent currents (T-currents) in human Ca_v_3.2-transfected HEK293 cells. In Ca_v_3.2-expressing HEK293 cells superfused with the extracellular solution, T-currents were determined every 12 s by whole-cell patch-clamp recordings; a test pulse at −20 mV (200 ms duration) from a holding potential at −80 mV. After the T-currents became stable, the cells were challenged with vehicle (A) or Na_2_S at 10 mM (B–E) for 10 min in the absence (A, B) or presence of THGP at 1 (C), 3 (D) or 10 mM (E) which was applied 5 min before Na_2_S challenge. (F, G) Effects of THGP on the Na_2_S-induced fold increase in T-currents (F), and on the baseline T-currents (G). V, vehicle. Data show the mean with S.E.M from 4 to 5 different cells. **P < 0.01 vs. V + V; †P < 0.05, ††P < 0.01 vs. V + Na_2_S.

The effect of THGP on sulfide-induced enhancement of Ca_v_3.2 T-channel activity was examined in HEK293 cells that stably express human Ca_v_3.2 channels, using a whole-cell patch-clamp technique. After the confirmation of stable T-channel-dependent currents (T-currents), the cells were stimulated with Na_2_S at 10 μM almost doubled T-currents, an effect persisting for at least 10 min (Fig. 4A and B), as reported previously [23]. THGP at 1–10 mM prevented the Na_2_S-induced enhancement of T-currents in a concentration-dependent manner (Fig. 4C–F), whereas THGP alone in the same concentration range did not alter the baseline T-currents (Fig. 4C–E, G).

### 3.4 Inhibitory effect of THGP on the Ca_v_3.2-dependent pain induced by exogenously applied sulfide in mice

**Fig. 5:**
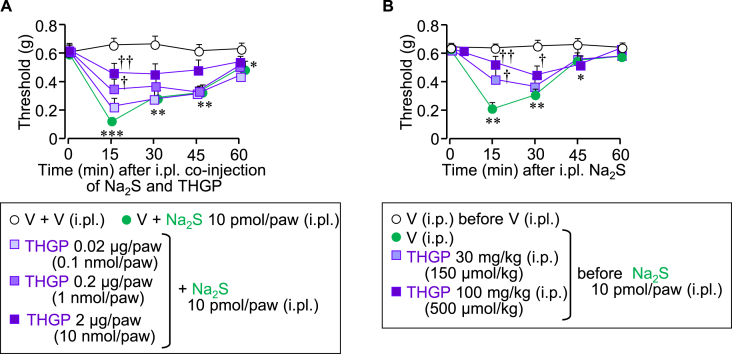
Effect of local or systemic administration of THGP on the mechanical allodynia induced by intraplantar (i.pl.) injection of Na_2_S in mice. Nociceptive threshold in the ipsilateral hindpaw before and after intraplantar (i.pl.) injection of Na_2_S at 10 pmol/paw in mice was assessed repeatedly by von Frey test. (A) Mice received i.pl. co-injection of THGP at 0.02, 0.2 or 2 μg/paw (0.1, 1 or 10 nmol/paw, respectively) and Na_2_S in mice. (B) Mice received i.p. administration of THGP at 30 or 100 mg/kg (150 or 500 μmol/kg), 30 min before i.pl. injection of Na_2_S in mice. V, vehicle. Data show the mean ± S.E.M. for 5 mice (A), 10–12 mice (B). *P < 0.05, **P < 0.01, ***P < 0.001 vs. V + V (A) or V before V (B); †P < 0.05, ††P < 0.01 vs. V + Na_2_S (A) or V before Na_2_S (B).

Given the direct interaction between THGP and sulfide *in vitro*, as shown above, we tested whether THGP could suppress the sulfide-induced pain, in which Ca_v_3.2 plays an essential role [18,20,23]. Intraplantar (i.pl.) injection of Na_2_S at 10 pmol/paw caused mechanical allodynia in mice, an effect suppressed by co-injection of THGP at 0.02–2 μg/paw (0.1–10 nmol/paw) in a dose-dependent manner (Fig. 5A). Systemic (i.p.) preadministration of THGP at 30 or 100 mg/kg (150 or 500 μmol/kg, respectively) also reduced the i.pl. Na_2_S-induced allodynia (Fig. 5B).

### 3.5 Inhibitory effect of THGP on Ca_v_3.2-dependent visceral pain caused by endogenous sulfide in mice

**Fig. 6:**
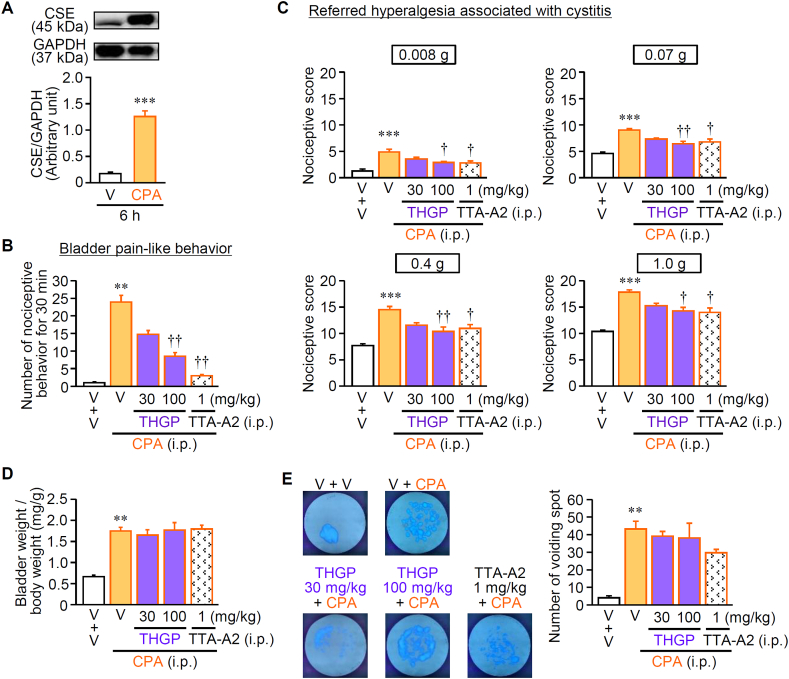
Effect of systemic administration of THGP on the bladder pain associated with cyclophosphamide (CPA)-induced cystitis. Mice received i.p. administration of CPA at 400 mg/kg, and thereafter, i.p. administration of THGP at 30 and 100 mg/kg or TTA-A2, a T-channel inhibitor, at 1 mg/kg, 3 h after CPA treatment. After the assessment of bladder pain/referred hyperalgesia and of micturition frequency (6 h after i.p. CPA), the bladder was excised from the sacrificed mice to perform bladder weight measurement and Western blotting. (A) Upregulation of cystathionine-γ-lyase (CSE), an H_2_S-forming enzyme, in the mouse bladder 6 h after CPA treatment. The top pictures show the typical photographs of Western blotting, and bottom graphs indicate the quantified data by densitometry. (B) Effects of THGP and TTA-A2 on the bladder pain-like nociceptive behavior observed for 30 min starting 3.5 after i.p. CPA. (C) Effects of THGP and TTA-A2 on the referred hyperalgesia evaluated by von Frey tests 4 h after i.p. CPA. (D) Lack of effects of THGP and TTA-A2 on the increased bladder weight 6 h after i.p. CPA. (E) Lack of effects of THGP and TTA-A2 on the increased micturition frequency for 2 h starting 4 h after i.p. CPA. Typical photographs of visualized voiding spots and the number of voiding spots are shown. Data show the mean with S.E.M. for 5–7 mice. V, vehicle. **P < 0.01, ***P < 0.001 vs. V + V; †P < 0.05, ††P < 0.01 vs. V + CPA.

**Fig. 7:**
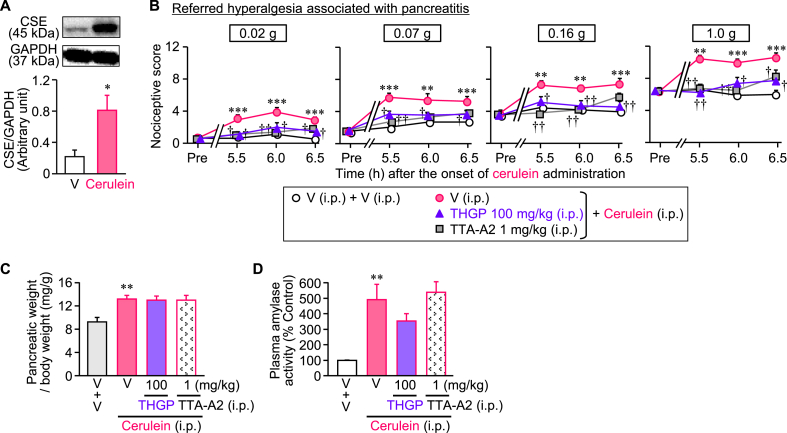
Effect of systemic administration of THGP on the pancreatic pain associated with cerulein-induced pancreatitis in mice. Mice received repeated i.p. injection of cerulein at 50 μg/kg at 1-h intervals, 6 times in total. THGP at 100 mg/kg or TTA-A2 at 1 mg/kg was administered i.p. 5 min after the final (6th) cerulein injection, and the referred hyperalgesia was repeatedly evaluated 5.5, 6 and 6.5 h after the onset of repeated cerulein injections (i.e. 0.5, 1 and 1.5 h after the final cerulein injection). Seven hours after the onset of repeated cerulein injections, the blood was withdrawn from the anesthetized mice for the assessment of plasma amylase activity, and the pancreatic tissue was excised from the sacrificed mice afterwards, to perform pancreatic weight measurement and Western blotting. (A) Upregulation of cystathionine-γ-lyase (CSE), an H_2_S-forming enzyme, in the mouse pancreas 7 h after the onset of repeated cerulein injections. The top pictures show the typical photographs of Western blotting, and bottom graphs indicate the quantified data by densitometry. (B) Effects of THGP and TTA-A2 on the referred hyperalgesia evaluated repeatedly by von Frey test 5.5–6.5 h after the onset of repeated cerulein injections. (C, D) Lack of effect of THGP and TTA-A2 on the increased pancreatic weight (C) and plasma amylase activity (D) in cerulein-treated mice. V, vehicle. Data show the mean with S.E.M. from 9 mice. **P < 0.01, ***P < 0.001 vs. V + V; †P < 0.05, ††P < 0.01 vs. V + cerulein.

Endogenous sulfide/H_2_S generated from l-cysteine by the upregulated CSE participates in Ca_v_3.2-dependent visceral pain associated with CPA-induced cystitis and cerulein-induced pancreatitis in mice [19,[29], [30], [31]]. A single systemic (i.p.) administration of CPA induced upregulation of CSE protein in the bladder tissue (Fig. 6A), bladder pain-like nociceptive behavior (Fig. 6B), referred hyperalgesia in the skin region between the anus and urethral opening (Fig. 6C), bladder swelling (increased bladder weight) (Fig. 6D) and increased urinary frequency (increased voiding spots) (Fig. 6E), as reported elsewhere [29,30]. THGP at 100 mg/kg as well as TTA-T2, a selective T-channel blocker, at 1 mg/kg, when administered i.p. 3 h after CPA administration, significantly reduced the nociceptive behavior and referred hyperalgesia (Fig. 6B and C), but not bladder swelling or increased urinary frequency (Fig. 6D and E). Cerulein at 50 μg/kg, repeatedly administered i.p. at 1-h intervals, 6 times in total, caused upregulation of CSE protein in the pancreatic tissue (Fig. 7A), referred hyperalgesia in the upper abdomen (Fig. 7B), pancreatic swelling (increased pancreatic weight) (Fig. 7C) and increased plasma amylase levels (Fig. 7D), in agreement with our previous study [19]. THGP at 100 mg/kg as well as TTA-A2 at 1 mg/kg, administered i.p. 5 min after the final injection of cerulein, significantly suppressed the referred hyperalgesia (Fig. 7B), but not pancreatic swelling (Fig. 7C) or increased plasma amylase activity (Fig. 7D). Thus, THGP capable of trapping sulfide and the T-channel inhibitor suppressed visceral pain accompanying cystitis and pancreatitis, without affecting tissue swelling or damage, being consistent with the previous findings that CSE inhibitors prevented visceral pain in the same cystitis and pancreatitis models [19,29].

## 4 Discussion

**Fig. 8:**
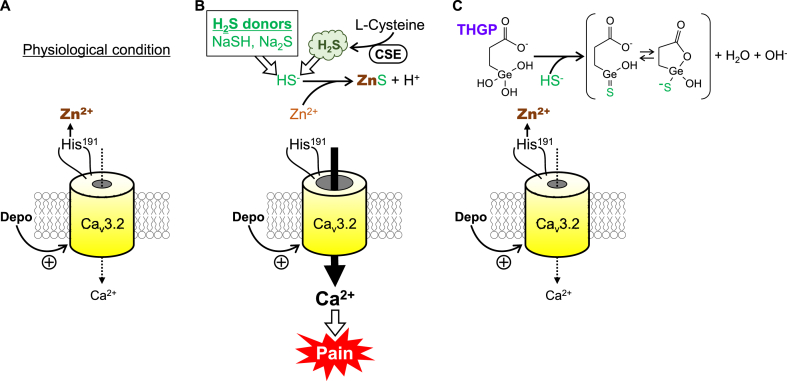
Presumed mechanisms by which THGP inhibits the sulfide-induced enhancement of Ca_v_3.2 activity and pain. (A) In the physiological conditions, the Ca_v_3.2 channel activity is suppressed in part by Zn^2+^ linked via coordinate bonding to His^191^ of the extracellular domain of Ca_v_3.2 expressed in the peripheral endings of primary sensory neurons. (B) Sulfide such as HS^−^, derived from exogenously applied H_2_S donors or from endogenous H_2_S produced by cystathionine-γ-lyase (CSE) from l-cysteine, interacts with Zn^2+^ linked to the His^191^, leading to cancellation of the Zn^2+^ inhibition of Ca_v_3.2 and acceleration of pain sensation. (C) THGP directly reacts with HS^−^ and forms a sulfur-containing reaction product, thereby inhibiting the sulfide-induced pain. CSE, cystathionine-γ-lyase; Depo, depolarization.

Under physiological conditions, the activity of Ca_v_3.2 T-channels is attenuated in part by Zn^2+^ linked via coordinate bonding to a nitrogen atom in the imidazole ring of His^191^ present in the second extracellular loop of domain I of Ca_v_3.2 (Fig. 8A), and the Zn^2+^ inhibition of Ca_v_3.2 can be reversed by Zn^2+^ chelators or other Zn^2+^-sensitive compounds including thiols such as l-cysteine [25,26]. Considering that sulfides, but not polysulfides, easily react with Zn^2+^ and produce ZnS [47,48], HS^−^ derived from endogenous H_2_S generated by CSE or other enzymes and from exogenously applied H_2_S donors is considered to interact with Zn^2+^ linked by coordinate bonding to His^191^ of Ca_v_3.2 and cancel the Zn^2+^ inhibition of Ca_v_3.2 activity (Fig. 8B). The present study demonstrated that THGP, the hydrolysate of Ge-132, directly reacted with HS^−^ and formed a reaction product containing a sulfur atom binding to the germanium atom of THGP (Fig. 1A), thereby interfering the interaction of HS^−^ with Zn^2+^ linked via coordinate bonding to His^191^ of Ca_v_3.2 and consequently inhibiting the sulfide-induced enhancement of Ca_v_3.2 activity (Fig. 8C).

The acceleration of Ca_v_3.2 activity by sulfide/H_2_S, particularly generated by CSE, is involved in a number of pathological pain including visceral pain accompanying cystitis or pancreatitis [19,[29], [30], [31]], as shown in the present study, and also somatic inflammatory and neuropathic pain [17,27,28]. In this context, THGP might be useful to treat a wide variety of intractable somatic pain, in addition to bladder and pancreatic pain. Our findings from ^1^H NMR analysis that the reaction between 10 mM THGP and 10 mM Na_2_S decreased by nearly half in the presence of 3 mM ZnCl_2_ (see Fig. 3) suggest that about 3-fold higher concentrations of THGP than Zn^2+^ might be required to halve the interaction between Zn^2+^ and sulfide. Since serum zinc concentrations are around 15 μM in the mammalian body [49], it is estimated that the effective blood concentrations of THGP to halve the sulfide-Zn^2+^ binding are around 45 μM. It has been reported that the serum THGP concentration reaches around 20 μM (4 μg/mL) at a peak time, 3–6 h after oral administration of Ge-132 at 100 mg/kg in rats [45]. Therefore, the estimated effective blood concentration, 45 μM, of THGP is considered achievable after i.p. administration of THGP at 100 mg/kg that inhibited sulfide-dependent somatic and visceral pain in the present study (see Fig. 5, Fig. 6, Fig. 7). We consider that Zn^2+^ and THGP would react with sulfide in a competitive manner at least in a solution state, which is also associated with solubility equilibrium of ZnS in a biological fluid.

H_2_S is now known as a multifunctional gasotransmitter along with NO and CO, and exhibits a variety of biological activity through its interaction with metals binding to proteins including heme proteins, antioxidant activity, sulfhydration of cysteine residues present in proteins, and others [50]. It is likely that these biological activities of H_2_S is also inhibited by THGP. Sulfide/H_2_S interacts with several metals, such as zinc, iron, copper and nickel [50], whereas the effects of THGP on the interaction of sulfide with metals other than zinc in the mammalian body have yet to be examined.

## 5 Conclusions

Our study demonstrates that THGP, a hydrolysate of Ge-132, directly reacts with sulfide, resulting in suppression of Ca_v_3.2-dependent pain caused by sulfide applied exogenously or generated endogenously. Therefore, Ge-132 may serve as a medicine for treatment of pathological pain. H_2_S, a toxic gas, at higher concentrations binds to cytochrome *c* oxidase in the electron transport chain, leading to cell death [50], and needs pharmacological interventions [51]. Such a toxic outcome of endogenous and inhaled H_2_S may also be protected by THGP. Endogenous sulfide/H_2_S appears to play a dual role, i.e. being pro-inflammatory and anti-inflammatory (protective), and long-term complete inhibition of H_2_S-generating enzymes may not necessarily be beneficial, particularly in the cardiovascular system [50,52]. In this context, Ge-132 may be useful to trap the excessive sulfide/H_2_S generated in pathological conditions and maintain appropriate blood sulfide/H_2_S levels.

## Author contribution

FS, YS, TN and AK designed this project. FS, NK, YS, KS, HM, HY, GT, SM, YK and MT performed the experiments. FS, NK, YS, KS, HM, TN, HY, GT, SM, YK, MT and AK analyzed data and discussed results. FS, NK and AK wrote the manuscript. TO and SY contributed materials and technical guiding.

## Declaration of competing interest

The authors declare that the research was conducted in the absence of any commercial or financial relationships that could be construed as a potential conflict of interest.

## Data Availability

Data will be made available on request.
