# Supplementary material for: A hydrolysate of poly-trans-[(2-carboxyethyl)germasesquioxane] (Ge-132) suppresses Cav3.2-dependent pain by sequestering exogenous and endogenous sulfide
Source: Redox Biol. 2022 Dec 15;59:102579. doi: 10.1016/j.redox.2022.102579 (PMC9800310; doi:10.1016/j.redox.2022.102579)
Supplement: Multimedia component 1 [file mmc1.pdf]

(A)

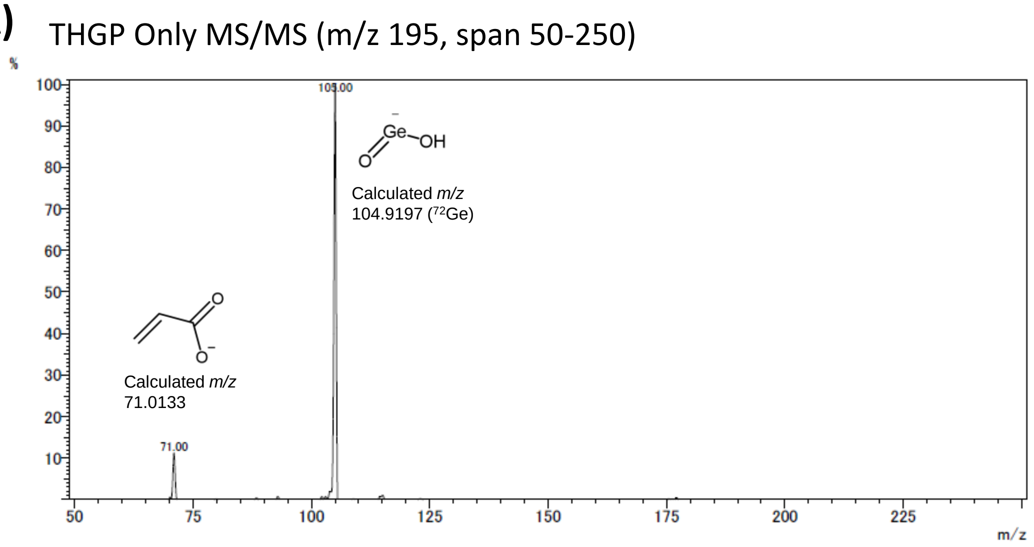

(B)

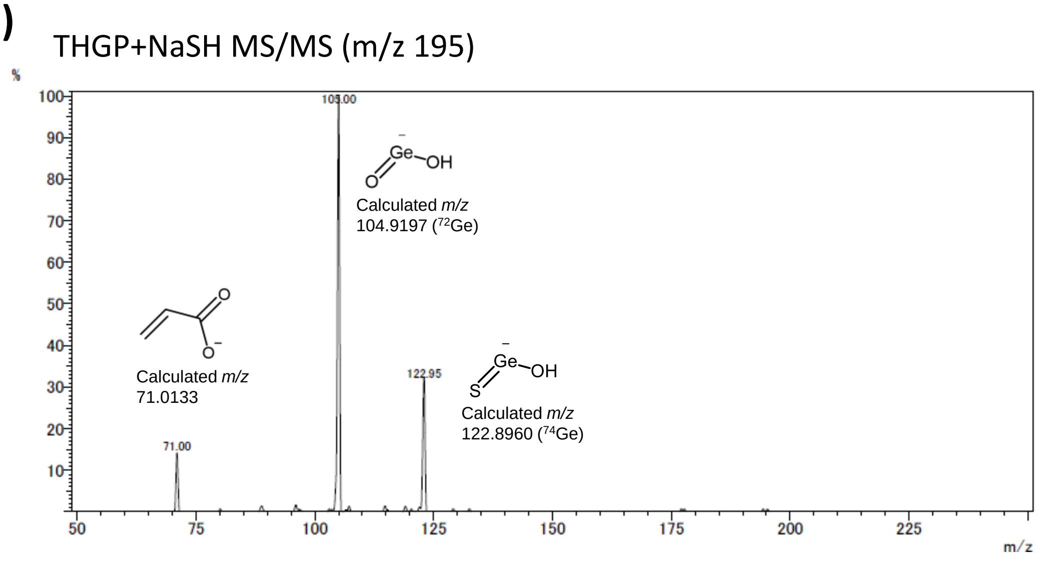

Fig. S1. Product ion mass spectra provided by MS/MS analysis of the precursor signals at *m/z* 195 in THGP at 1 mM alone (A) and in the mixture of THGP at 1 mM and NaSH at 1 mM (B) with a collision energy of -35 V.

Supplement Figure S1
